# APE1/Ref-1 redox-specific inhibition decreases survivin protein levels and induces cell cycle arrest in prostate cancer cells

**DOI:** 10.18632/oncotarget.23493

**Published:** 2017-12-13

**Authors:** David W. McIlwain, Melissa L. Fishel, Alexander Boos, Mark R. Kelley, Travis J. Jerde

**Affiliations:** ^1^ Department of Pharmacology and Toxicology, Indiana University School of Medicine, Indianapolis, Indiana, USA; ^2^ Department of Pediatrics, Herman B Wells Center for Pediatric Research, Indiana University School of Medicine, Indianapolis, Indiana, USA; ^3^ Department of Biochemistry and Molecular Biology, Indiana University School of Medicine, Indianapolis, Indiana, USA

**Keywords:** prostate cancer, APE1/Ref-1, survivin, NFκB signaling, redox regulation

## Abstract

A key feature of prostate cancer progression is the induction and activation of survival proteins, including the Inhibitor of Apoptosis (IAP) family member survivin. Apurinic/apyrimidinic endonuclease 1/redox effector factor 1 (APE1/Ref-1) is a multifunctional protein that is essential in activating oncogenic transcription factors. Because APE1/Ref-1 is expressed and elevated in prostate cancer, we sought to characterize APE1/Ref-1 expression and activity in human prostate cancer cell lines and determine the effect of selective reduction-oxidation (redox) function inhibition on prostate cancer cells *in vitro* and *in vivo*. Due to the role of oncogenic transcriptional activators NFĸB and STAT3 in survivin protein expression, and APE1/Ref-1 redox activity regulating their transcriptional activity, we assessed selective inhibition of APE1/Ref-1’s redox function as a novel method to halt prostate cancer cell growth and survival. Our study demonstrates that survivin and APE1/Ref-1 are significantly higher in human prostate cancer specimens compared to noncancerous controls and that APE1/Ref-1 redox-specific inhibition with small molecule inhibitor, APX3330 and a second-generation inhibitor, APX2009, decreases prostate cancer cell proliferation and induces cell cycle arrest. Inhibition of APE1/Ref-1 redox function significantly reduced NFĸB transcriptional activity, survivin mRNA and survivin protein levels. These data indicate that APE1/Ref-1 is a key regulator of survivin and a potentially viable target in prostate cancer.

## INTRODUCTION

Prostate cancer (PCa) is one of the most common male malignancies and the third leading cause of cancer-related death of men in the United States [1–2]. Small prostatic carcinomas exist in up to 29% of men in their thirties and 64% of men in their sixties, with most of these carcinomas being indolent and/or cured by surgery or radiation [3–5]. However, some men develop an aggressive phenotype that metastasizes and becomes incurable once colonizing the bone [6–7]. These bone metastases produce osteoblastic lesions that are associated with high morbidity and high mortality [8] and attempts at delaying this tumor progression with chemotherapeutic agents have only prolonged survival a few months. [9–10] In order to create more effective treatments where conventional therapeutics have failed, a better understanding of the aggressive phenotype of the disease is of utmost importance and a great unmet medical need.

It is now well-established that reduction-oxidation (redox) regulation of critical transcriptional activators plays an essential role in cell proliferation and survival in a number of different cancers, including prostate cancer [11–13]. Apurinic/apyrimidinic endonuclease 1/redox factor 1 (APE1/Ref-1) is a multifunctional protein that participates in DNA repair and redox transcriptional regulation [14–15]. APE1/Ref-1 has been implicated in the development and progression of numerous cancer types, is conversely correlated to tumor radiation and chemotherapy sensitivity, and is overexpressed in prostate cancer [16–20]. APE1/Ref-1 redox regulation of transcriptional activators occurs through cysteine residues within the DNA binding or transactivation domain of the transcription factor and is essential for full activation of certain transcriptional activators including the oncogenic transcriptional activators AP-1, HIF-1α, NF-κB and STAT3. Treatment with small molecule inhibitors of the redox activity of APE1/Ref-1, such as APX3330 has been shown to diminish the activity of these redox-regulated transcriptional activators [21–23]. Furthermore, the blockade of APE1/Ref-1’s redox activity has been shown to reduce growth-promoting, inflammatory and anti-apoptotic activities in cells [24–25].

The ability of cancer cells to overcome apoptotic signals is crucial for tumor progression. Survivin is an Inhibitor of Apoptosis (IAP) family member, and it is overexpressed in prostate cancer. Survivin has been implicated in resistance to various chemotherapeutic and pro-apoptotic agents [26–28]. Survivin is classically known as an inhibitor of caspases due to its single BIR (Baculovirus IAP Repeat) domain, but recently survivin has been found to be crucial in cell cycle progression as a member of the chromosomal passenger complex (CPC) [29]. As part of the CPC, survivin along with members Borealin, INCENP and Aurora B kinase orient the chromosomes during mitosis. Previously, our lab has demonstrated that survivin is juxtaposed to inflammation in human prostate cancer specimens and may play a role in repair and recovery of prostatic tissue [30]. Attempts at directly targeting survivin have ultimately failed in clinic, therefore new approaches or therapeutics that in some way block the expression or function of survivin are needed.

Accumulating evidence demonstrates that APE1/Ref-1 is a key regulator of cancer cell growth and survival signaling and is upstream of pathways that regulate survivin expression [31]. Here, we report that inhibition of APE1/Ref-1 redox signaling activity decreases prostate cancer cell proliferation, decreases the transcriptional activity of NFκB, and downregulates survivin expression in prostate cancer cells *in vitro* and *in vivo*. This is the first report to our knowledge that mechanistically demonstrates that APE1/Ref-1 redox-specific inhibitors are a viable therapeutic option for prostate cancer treatment.

## RESULTS

### APE1/Ref-1 and survivin are nuclear and cytoplasmic localized in human prostate cancer

**Figure 1:**
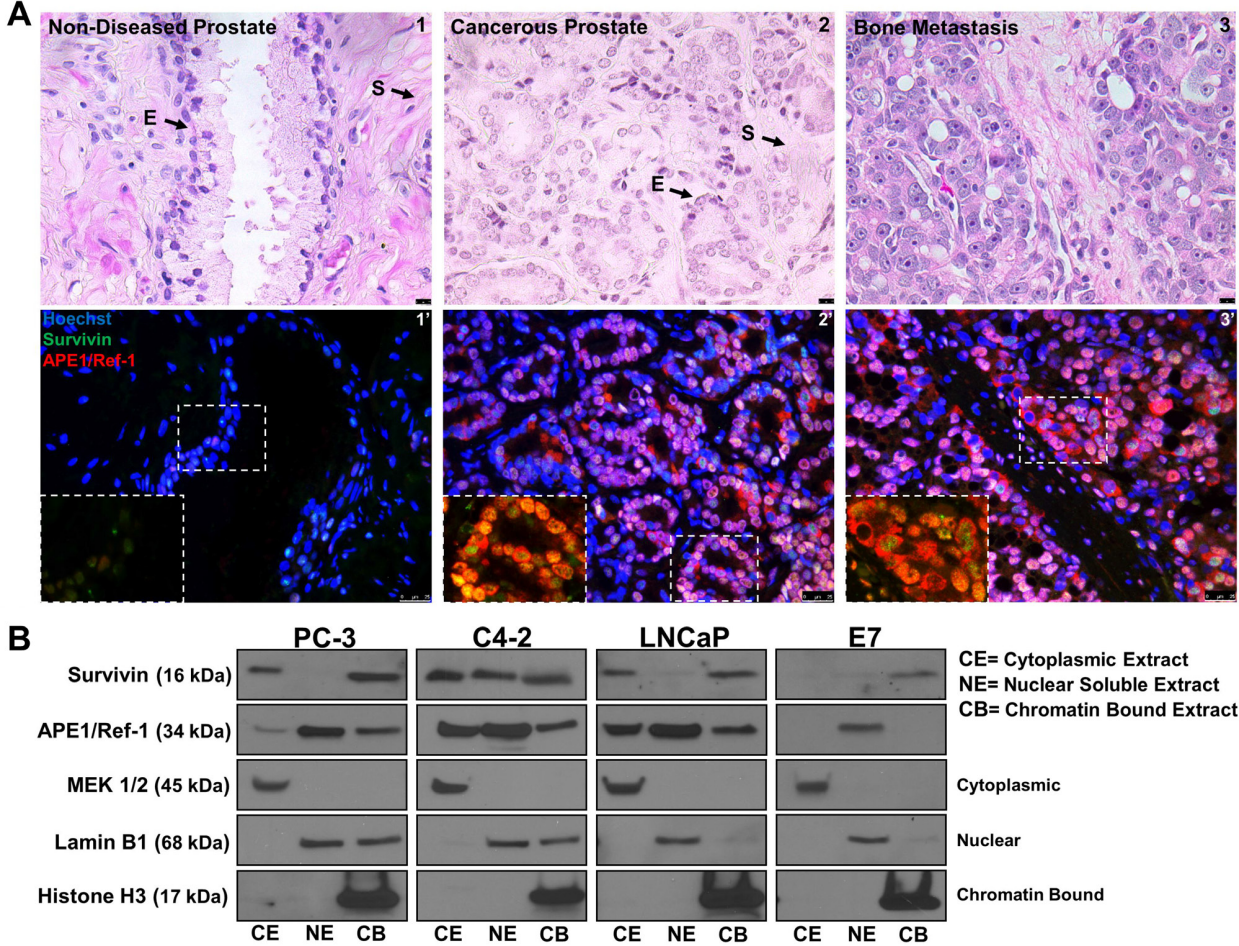
APE1/Ref-1 and survivin are nuclear and cytoplasmic localized in human prostate cancer (**A**) Hematoxylin and Eosin staining representing non-diseased (peripheral zone taken from cystoprostatectomy) and cancerous human prostate specimens (1–3). Scale bar = 10 µM. Immunofluorescent images of stained non-diseased and cancerous sections (1′-3′) for APE1/Ref-1 (red) and survivin (green). Scale bar = 25 µm, *n* = 12. (**B**) Cellular fractionation representing basal survivin and APE1/Ref-1 protein localization in cancerous (PC-3, C4-2 and LNCaP) and non-cancerous (E7) prostatic cell lines. MEK 1/2 (cytoplasmic), Lamin B1 (nuclear) and Histone H3 (chromatin bound) were used as controls for each subcellular fraction

To confirm APE1/Ref-1 and survivin protein expression in prostate cancer, we performed immunofluorescence using human non-diseased and cancerous prostate specimens (Figure 1A). We found that APE1/Ref-1 and survivin are expressed in non-diseased and cancerous prostate specimens (*n* = 12). Both proteins are universally expressed in bone metastasis. Localization of both proteins was primarily found to be nuclear and localized to the epithelium but in cancerous prostates cytoplasmic localization was observed (Figure 1A Inlet). To verify if well-characterized prostatic cell lines displayed the same expression pattern, PC-3, C4-2, LNCaP and non-cancerous E7 cells were fractionated into cytoplasmic, nuclear soluble and chromatin bound fractions and immunoblotting was performed evaluating APE1/Ref-1 and survivin protein localization (Figure 1B). E7 cell line is a normal prostatic epithelial cell line that was transformed using the human papillomavirus 16 (HPV16) E7 gene. MEK 1/2, Lamin B1 and Histone H3 were used as the respective controls for each fraction. APE1/Ref-1 protein localization was found to be in all three subcellular fractions in cancerous cell lines but only the nuclear soluble fraction in non-cancerous E7 cells. Survivin protein localization was primarily found in the cytoplasmic and chromatin bound fraction with some variable expression in the nuclear soluble fraction in the cancerous cell lines but localized only to the chromatin bound fraction in the non-cancerous E7 cells. This mirrors the expression pattern found in the human specimens. Additionally, APE1/Ref-1 and survivin protein levels were found to be significantly higher in PC-3, C4-2 and LNCaP cell lines compared to the E7 cell line (Supplementary Figure 1).

### APE1/Ref-1 redox inhibition decreases prostate cancer cell number

**Figure 2:**
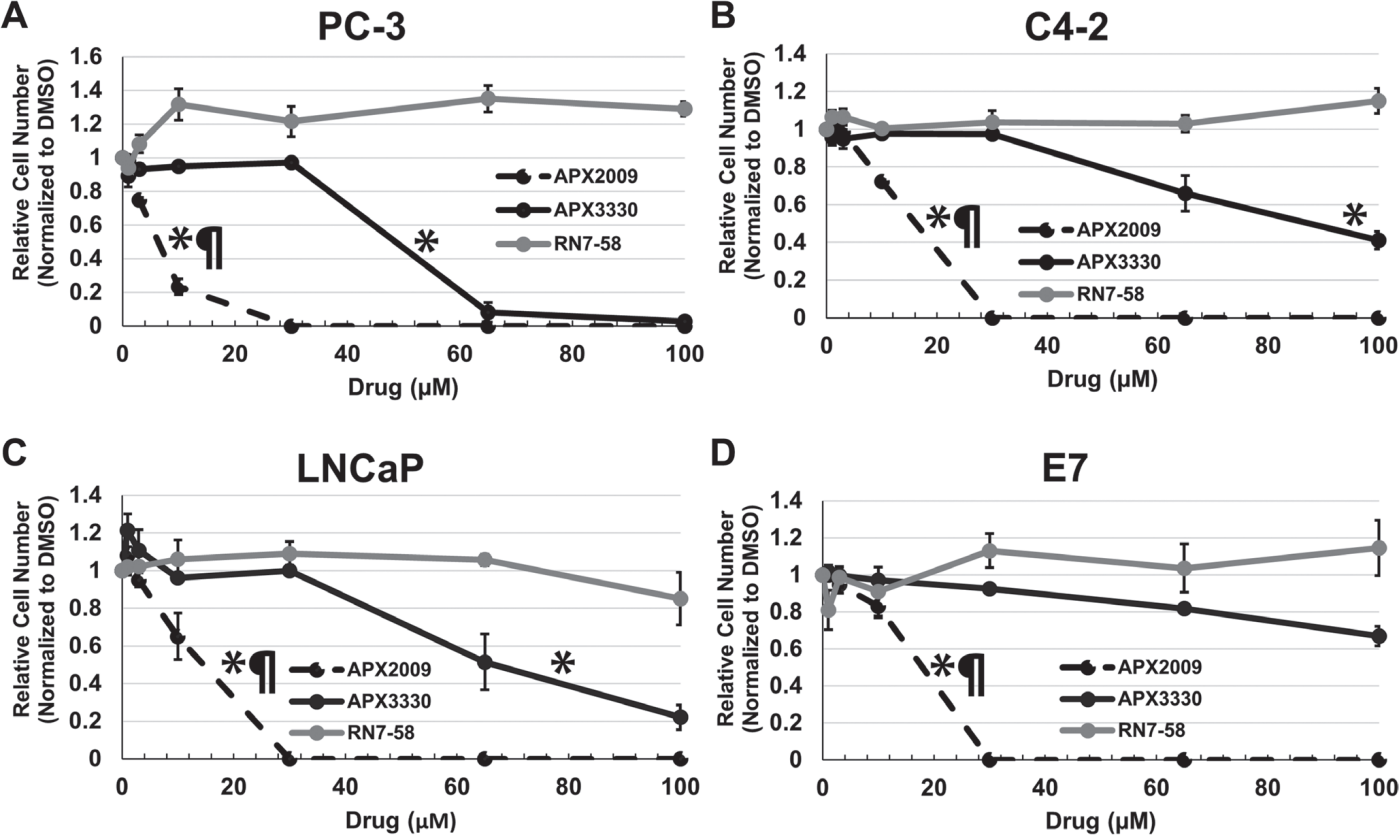
APE1/Ref-1 redox function specific inhibitors decrease cell number in a concentration dependent manner PC-3 (**A**), C4-2 (**B**), LNCaP (**C**) and E7 (**D**) cell lines were treated with increasing concentrations of redox-specific inhibitor APX3330, more potent analogue APX2009, and inactive analogue RN7-58 for 5 days. The cells were fixed and stained with methylene blue and measured via spectrophotometry. IC_25_ and IC_50_ were determined as the concentrations of drug at which there was a 25% and 50% reduction in absorbance compared to vehicle control (DMSO) and were used for subsequent experiments. *n* = 3. EC50s were compared between the drugs: ^*^ denotes *p* < 0.05 drug EC50 versus RN7-58, while ¶ denotes *p* < 0.05, APX3330 versus APX2009.

To determine if inhibition of APE1/Ref-1’s redox function affects cell number, prostatic cell lines were treated with increasing concentrations of APE1/Ref-1 redox-specific inhibitors APX3330 and APX2009 for five days and cell number was measured via methylene blue assay (Supplementary Figure 2). RN7-58 is an inactive analogue of the APX3330 and APX2009 chemical families and was used as a negative control. It has been shown to have no effect on APE1/Ref-1 redox function. [32] APX3330 and APX2009 inhibited cell number in a concentration-dependent manner (Figure 2A–2D). Growth IC_25_’s and IC_50_’s were determined (Table 1). Student’s *t*-test was performed to verify statistical IC_25_ and IC_50_ differences between APX3330 and APX2009. RN7-58 caused variable cell growth between cell lines but did not contain IC_25_ or IC_50_ drug concentrations. APX2009 was found to be 5–10 fold more efficacious than parent compound APX3330 in inhibiting cell proliferation. DMSO control was not significantly different from untreated cells.

**Table 1:** Growth IC_25_ and IC_50_’s were determined for each cell line using the 3 growth curves for APX3330 and APX2009

		APX3330 (µM)	APX2009 (µM)	*P* value
PC-3	IC_25_	36.0 +/− 1.0	2.2 +/− .5	> 0.0001
	IC_50_	54.7 +/− 1.6	8.9 +/− .7	> 0.0001
C4-2	IC_25_	57.4 +/− 3.8	7.6 +/− .2	0.0002
	IC_50_	89.5 +/− 7.8	14.2 +/− .3	0.0006
LNCaP	IC_25_	43.8 +/− 4.2	6.3 +/− 1.6	0.0011
	IC_50_	71.9 +/− 7.2	13 +/− 1.2	0.0013
E7	IC_25_	82.7 +/− 8.7	9.2 +/− .7	0.0011
	IC_50_	N/A	16.1+/− .8	-

Data are presented as mean ± s.e.m. *P* value was determined by comparing IC_25_ or IC_50_ values for APX3330 to that of APX2009 averages from the three separate determinations by unpaired Student’s t-test in each cell line.

### APE1/Ref-1 redox-specific inhibitors decrease survivin protein levels

**Figure 3:**
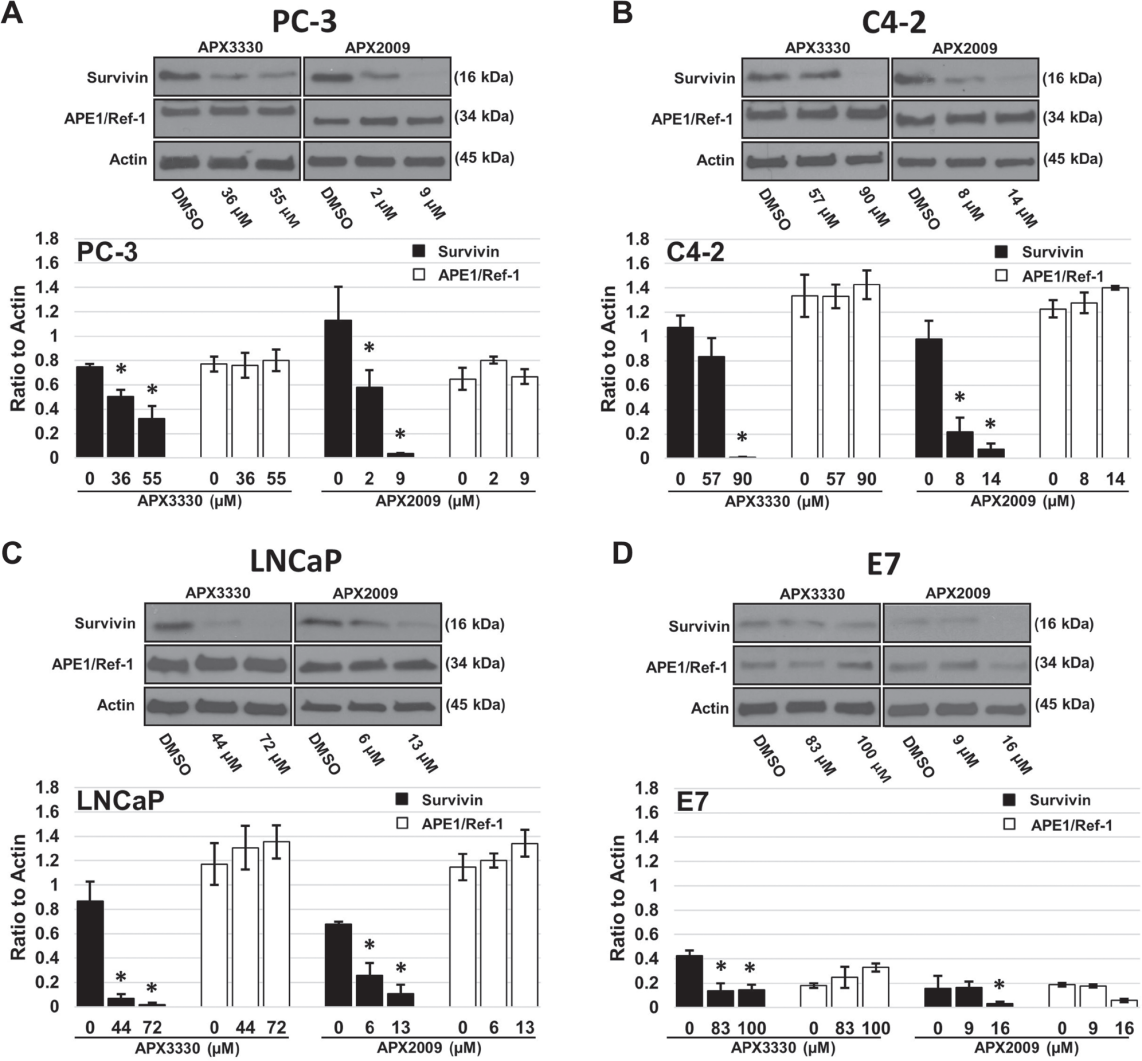
Treatment with APX3330 and APX2009 decreases survivin protein levels PC-3 (**A**), C4-2 (**B**), LNCaP (**C**) and E7 (**D**) cell lines were treated with DMSO, or the growth inhibitory IC_25_ and IC_50_ drug concentrations of APX3330 or APX2009 for 48 hours. Immunoblotting for survivin, APE1/Ref-1 and Actin as labeled. Data presented are representative of three determinations with densitometry quantification, *n* = 3, ^*^-denoting *p* < 0.05 (DMSO vs. IC_25_ and IC_50_ Drug Concentrations) as assessed by ANOVA.

Survivin plays an important role in prostate cancer cell proliferation and survival. Since survivin is controlled by APE1/Ref-1-regulated transcription factors in other organ systems such as the pancreas and liver [33–34], we hypothesized that treatment with APE1/Ref-1 redox-specific inhibitors APX3330 and APX2009 would decrease survivin protein levels, at least partially explaining the reduction in proliferative capacity. Prostate cancer cells treated with the respective growth inhibitory IC_25_ and IC_50_ drug concentrations of APX3330 and APX2009 (as determined in Table 1) exhibited a significant decrease in survivin protein expression within 48 hours compared to DMSO treated controls (Figure 3A–3D). In contrast, prostate cancer cell total APE1/Ref-1 protein levels were not significantly altered with treatment.

### APE1/Ref-1 siRNA reduces proliferation and survivin protein levels

**Figure 4:**
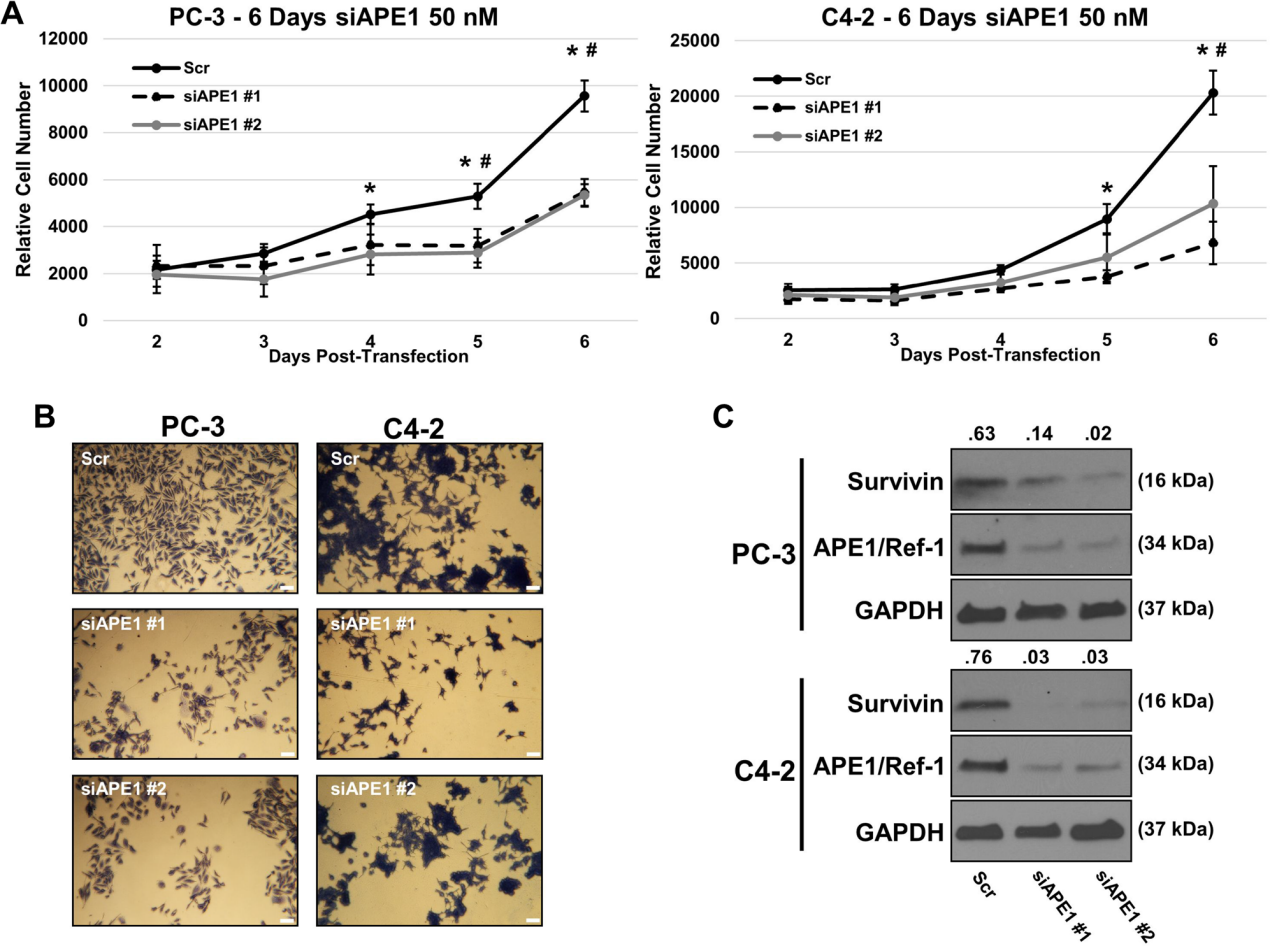
APE1/Ref-1 siRNA knockdown decreases cell proliferation and survivin protein levels (**A**) Separate aliquots of PC-3 and C4-2 cell lines were transfected with two distinct sequences of 50 nM APE1/Ref-1 siRNA (verified >70% knockdown by immunoblotting) and growth was compared to scrambled siRNA-transfected cells. *n* = 3, ^*^-denoting *p* < 0.05 within ANOVA (Scr vs siAPE#1), #- denoting *p* < 0.05 within ANOVA (Scr vs siAPE#2). (**B**) Representative pictures of fixed and methylene blue stained C4-2/PC-3 scrambled siRNA (Scr), survivin siRNA #1 (siAPE1 #1) and #2 (siAPE1 #2). (**C**) Immunoblotting was performed using antibodies for APE1/Ref-1, survivin and GAPDH as labeled after 72 hours post-transfection.

Using siRNA specific to APE1/Ref-1, we investigated if APE1/Ref-1 knockdown reduces cell growth and survivin protein levels. PC-3 and C4-2 cell lines were transfected with two distinct sequences of 50 nM APE1/Ref-1 siRNA (verified > 70% knockdown by immunoblotting) and growth was compared to scrambled siRNA-transfected cells (Figure 4A). Those cells transfected with APE1/Ref-1 siRNA grew at a significantly slower rate compared to those cells transfected with the scrambled siRNA. Representative pictures of fixed and methylene blue stained C4-2 and PC-3 scrambled siRNA (Scr), survivin siRNA #1 (siAPE1 #1) and #2 (siAPE1 #2) were taken (Figure 4B). Immunoblotting was performed 72 hours post transfection and survivin protein levels were found to be decreased compared to scrambled control (Figure 4C).

### Treatment with APX2009 induces G1 cell arrest but not cell death

**Figure 5:**
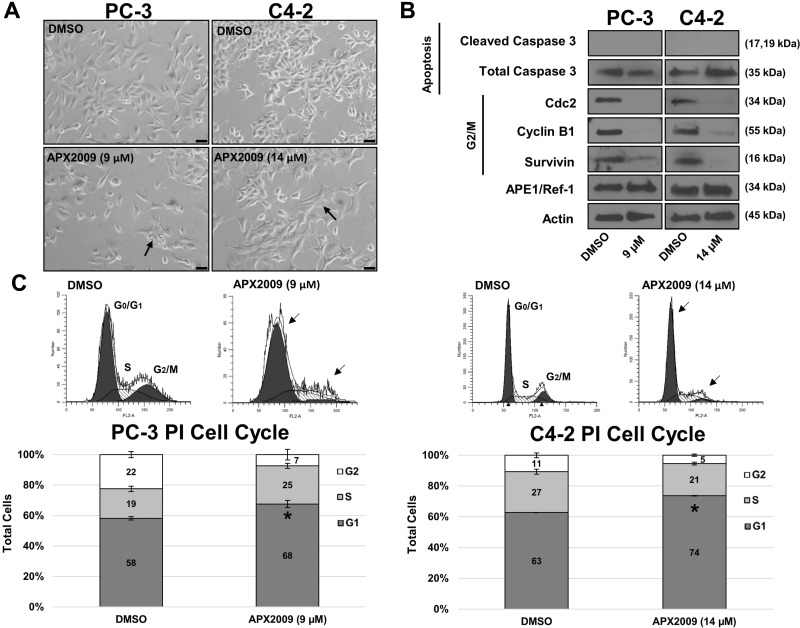
APE1/Ref-1 redox inhibition induces G1 cell arrest (**A**) PC-3 and C4-2 cell lines were treated with DMSO or APX2009 (9 and 14 µM, respectively) for 48 hours. Representative images were taken at 20× Magnification. Scale bar = 50 µm. (**B**) Immunoblotting was performed and membranes were probed with antibodies for Cleaved Caspase 3, Total Caspase, Cyclin B1, Cdc2, survivin and Actin as labeled. (**C**) PC-3 and C4-2 cells were treated with DMSO or APX2009 (9 and 14 µM, respectively) for 48 hrs and then collected and stained with RNAse/PI wash. Flow Cytometry was then performed. *n* =3, ^*^-denoting *p* < 0.05 by unpaired Student’s *t*-test.

Based on the increased potency of APX2009 over APX3330 in PCa cells, we focused on the second generation compound, APX2009 for the remainder of our molecular studies. To determine if inhibition of APE1/Ref-1 via APX2009 results in cell death due to loss of survival signaling, PC-3 and C4-2 cells were treated with either vehicle (DMSO) or previously-determined IC_50_ concentrations of APX2009 (9 µM in PC3 and 14 µM in C4-2) for 48 hours (Figure 5A) and cell lysates were collected for immunoblotting (Figure 5B). After APX2009 treatment, both PC-3 and C4-2 cells displayed an altered, flattened cellular morphology. However, treatment with these compounds did not induce cell death as determined by both a lack of increased caspase 3 cleavage (Figure 5B) and TUNEL labeling (data not shown). Because no increase in apoptosis was detected and cell cycle proteins Cdc2 and Cyclin B1 were dramatically decreased by APE1/Ref-1 inhibition (undetectable in PC-3 cells and a ∼97% decrease in C4-2 cells), cell cycle analysis was performed using Propidium Iodide (PI) staining. PC-3 and C4-2 cells were treated with APX2009 (9 µM and 14 µM, respectively) for 48 hours, stained with PI, and analyzed by flow cytometry (Figure 5C). We found that the percentage of cells in G1 significantly increased, *p* ˂ 0.05 via Student’s *t*-test, from 58 to 68% and 63 to 74% in PC3 and C4-2 cells, respectively, indicating G1 arrest of prostate cancer cells in response to APE1/Ref-1 inhibition. These effects on the cell cycle progression are similar to other recent reports of APE1/Ref-1 redox inhibition in cancer [35–36].

### APX2009 reduces survivin mRNA expression and perturbs NFκB activity

**Figure 6:**
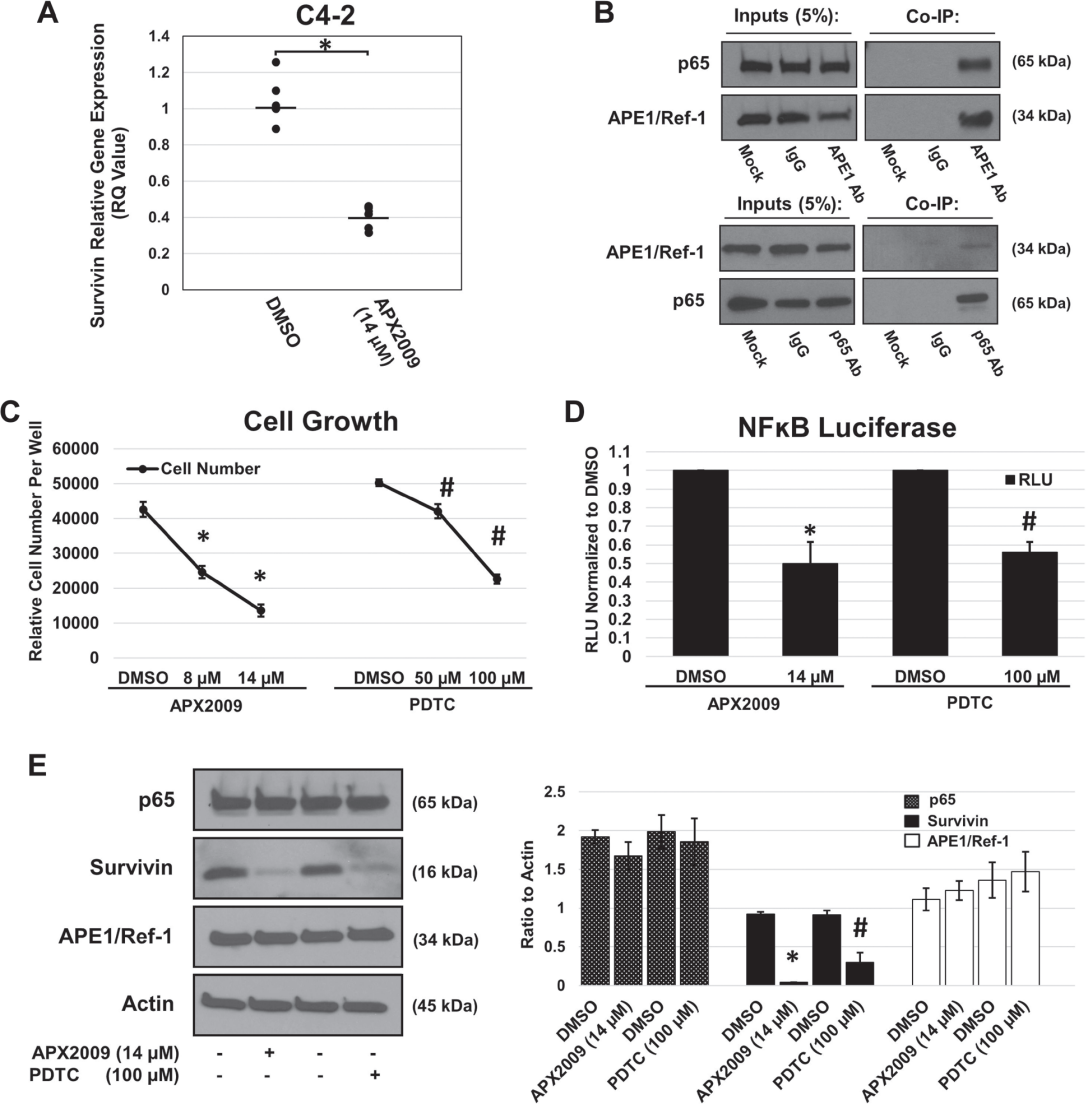
APE1/Ref-1 redox inhibition decreases survivin protein levels via NFκB (**A**) C4-2 cell line was treated with DMSO or APX2009 (14 µM) for 12 hours. RNA was isolated and RT-PCR for survivin was performed with HPRT1 as the reference gene. *n* = 6, ^*^-denoting *p* < 0.05 by unpaired Student’s *t*-test. (**B**) Immunoblot validation of APE1/Ref-1 and p65 Co-Immunoprecipitation (Co-IP) reactions. A 5% sample of the total input of each reaction (Input) and the total IP reaction (IP) were loaded for each reaction. Beads lacking a conjugated antibody (Mock) and generic IgG (IgG) were used as negative controls for each IP experimental reaction, APE1 antibody (top blots) and p65 (bottom blots). (**C**) C4-2 cell line was treated with DMSO, APX2009 (8 and 14 µM) or PDTC (50 and 100 µM) for 72 hours and cells were fixed and methylene blue was performed. *n* = 3, ^*^-denoting *p* < 0.05 (DMSO vs 8 and 14 µM APX2009) and #-denoting *p* < 0.05 (DMSO vs. 50 and 100 µM PDTC) as assessed by ANOVA. (**D**) C4-2 cells were transfected with NFκB–Luc construct and co-transfected with a Renilla vector, pRL-TK. After 16 hours, cells were treated with growth inhibitor IC_50_ concentrations of APX2009 and PDTC for 24 hours, and Firefly and Renilla luciferase activities were assayed using Renilla luciferase activity for normalization. All transfection experiments were performed in triplicate and repeated 3 times in independent experiments. Data are expressed as Relative Luciferase Units (RLU) normalized to DMSO showing the mean ± SEM. *n* = 3, ^*^-denoting *p* < 0.05 (DMSO vs. 14 µM APX2009) and #-denoting *p* < 0.05 (DMSO vs. 100 µM PDTC) within unpaired Students *t*-test. (**E**) C4-2 cell line was treated with APX2009 (14 µM) and NFĸB-selective inhibitor PDTC (100 µM) for 48 hours. Immunoblotting was performed with antibodies for survivin, p65, APE1/Ref-1 and Actin as labeled. Data presented are representative of three determinations with densitometry quantification, *n* = 33, ^*^-denoting *p* < 0.05 (DMSO vs. 14 µM APX2009) and #-denoting *p* < 0.05 (DMSO vs. 100 µM) as assessed by unpaired Student’s *t*-test.

Based on the observation that inhibition of APE1/Ref-1 reduces survivin protein levels, we sought to determine the mechanism by which APE1/Ref-1 regulates survivin expression, and ultimately, cell growth. We hypothesized that APE1/Ref-1’s redox control of transcription factors like NFκB would decrease survivin transcript levels. C4-2 cells were treated with vehicle or APX2009 IC_50_ (14 µM) for 12 hours. RNA was collected and RT-qPCR was performed using a primer/probe set for survivin (BIRC5) and HPRT1 for the reference gene (Figure 6A) using the conditions suggested by the SuperScript III Platinum One-Step qRT-PCR System (Invitrogen). Survivin mRNA was significantly reduced upon treatment with the relative quantity (RQ) value of ˂ 0.5. Survivin has been shown in other cancers to be regulated by NFκB, and NFĸB is regulated by APE1/Ref-1 redox signaling [37–40]. Therefore, we evaluated the ability of these two proteins to interact physically with each other. In Figure 6B, we demonstrate via co-immunoprecipitation that APE1/Ref-1 interacts with NFκB subunit p65 when using an APE1/Ref-1 antibody and in reverse experiments using a p65 antibody. To determine if NFκB signaling is responsible for cell growth and regulated by APE1/Ref-1 redox activity, we treated C4-2 cells with increasing concentrations of APX2009 and NFκB inhibitor ammonium pyrrolidinedithiocarbamate (PDTC) to determine the respective growth inhibition (Figure 6C). We then determined NFκB activity in the presence of these two drugs and found a significant two-fold decrease in NFκB-driven luciferase activity (Figure 6D). To further confirm a role of NFκB in regulating survivin protein levels, we treated C4-2 cells with 14 µM APX2009 and 100 µM PDTC for 48 hours and observed a significant 95% and 67% reduction in survivin protein levels, respectively (Figure 6E). In addition, we assessed the cellular localization of both NFκB and APE1/Ref-1 upon treatment with APX2009 (Supplementary Figure 3). p65 and APE1/Ref-1 were found to be co-localized in the nucleus however upon treatment with APX2009, p65 nuclear localization was diminished suggesting altered NFκB protein trafficking.

### APE1/Ref-1 redox inhibition decreases survivin protein levels and cell proliferation *in vivo*

**Figure 7:**
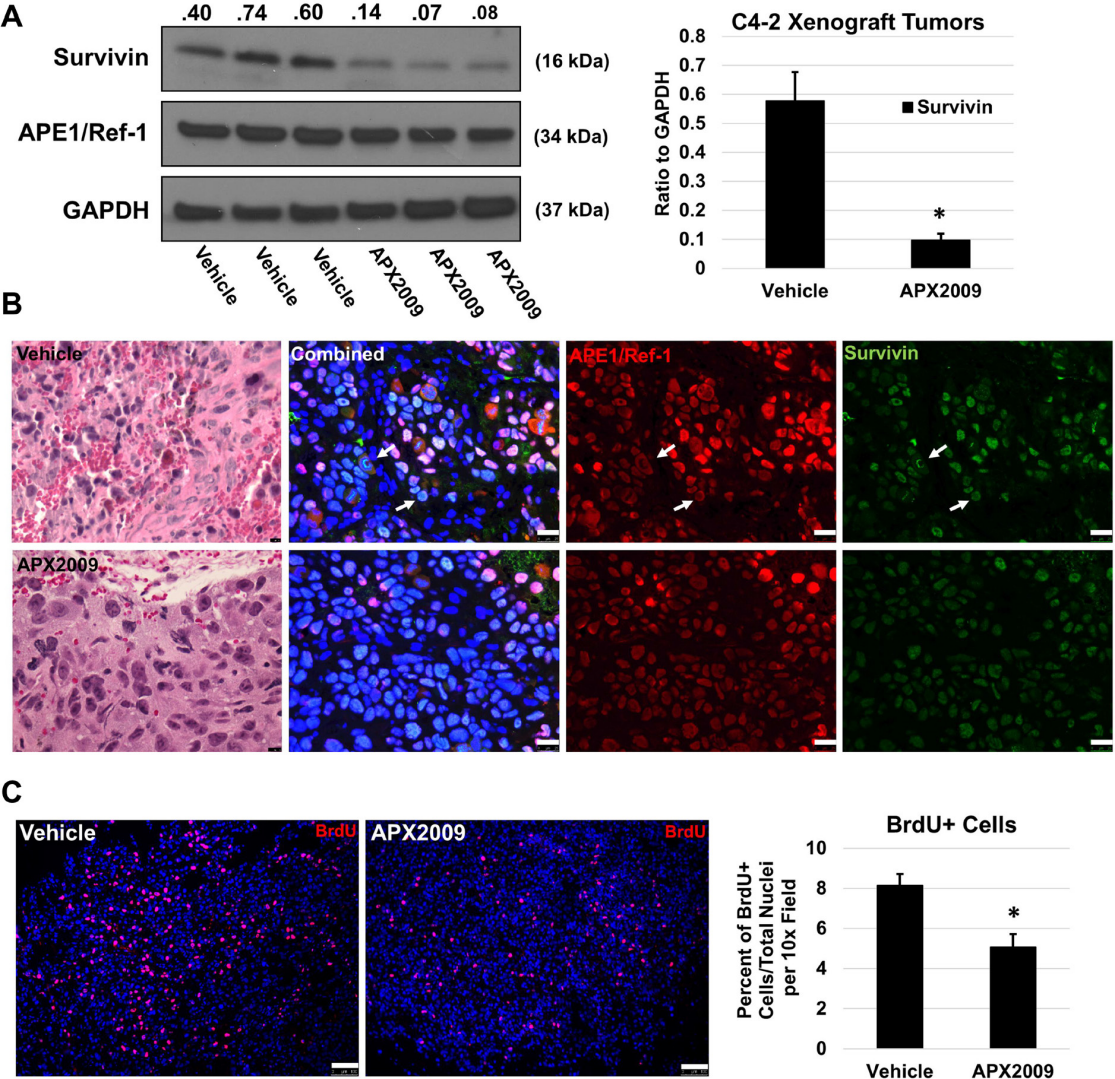
*In vivo* treatment with APX2009 reduces survivin protein levels and BrdU incorporation in C4-2 xenograft tumors C4-2 xenograft tumors were treated with Vehicle (Propylene Glycol Kolliphor HS15 Tween 80 (PKT)) or APX2009 (25 mg/kg, IP bid) for 5 days (*n* = 3). Tumors were removed and processed for either immunofluorescence or immunoblotting. (**A**) APE1/Ref-1 and survivin protein levels were measured using immunoblotting as labeled (Left). Data was presented graphically (Right), ^*^-denoting *p* < 0.05 by unpaired Student’s *t*-Test. (**B**) Hematoxylin and Eosin staining and immunofluorescence were performed using APE1/Ref-1 (red) and survivin (green) specific antibodies on vehicle and APX2009 groups. Representative images were taken. White arrows are depicting survivin nuclear staining patterns. Scale bar H&E = 10 µM. Scale bar immunofluorescence = 25 µm. (**C**) Mice were injected with BrdU 2 hours prior to sacrifice and tumors were collected and stained for BrdU incorporation (red). Scale bar = 100 µm. ImageJ Nucleus Counter was used to quantify number of BrdU+ nuclei and total nuclei per image. *n* = 3, ^*^-denoting *p* < 0.05 by unpaired Student’s *t*-test.

Based on the strong *in vitro* data demonstrating the regulation of survivin levels following APE1/Ref-1 inhibition, we confirmed our studies *in vivo* using C4-2 subcutaneous xenografts. The data in Figure 7 demonstrates that APE1/Ref-1 redox activity also plays a role in cell proliferation and survivin protein levels *in vivo*. Animals were treated with either APX2009 (25 mg/kg BID) or Vehicle for 5 days and then tumors were harvested. Total survivin protein via immunoblotting was significantly reduced (Figure 7A) when compared to control tumors. Survivin and APE1/Ref-1 localization via immunofluorescence remained nuclear with survivin co-localizing with the chromatin during mitosis (Figure 7B). Furthermore, BrdU incorporation was significantly reduced from 8.2% to 5.1% in the treatment group demonstrating that inhibition of APE1/Ref-1 redox activity reduces tumor cell proliferation (Figure 7C).

## DISCUSSION

**Figure 8:**
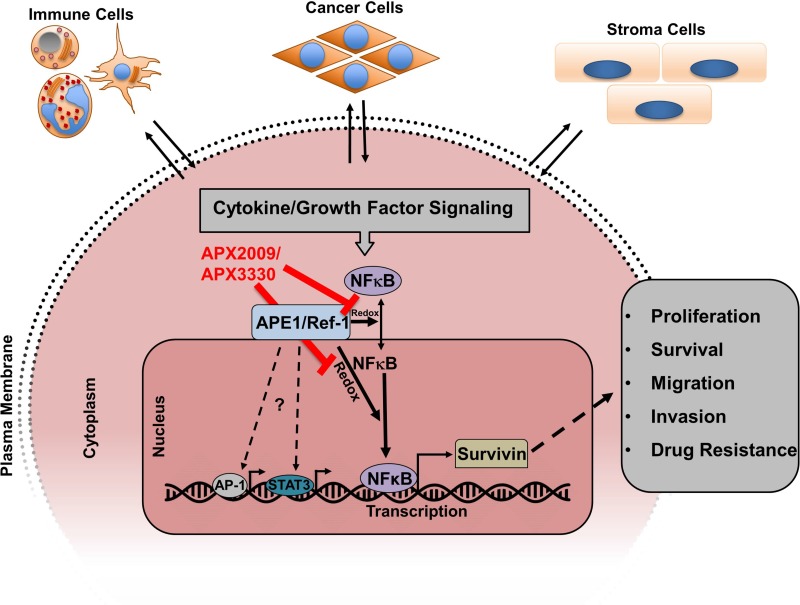
Model showing how cytokine/growth factor signaling induces survivin protein expression and where APX2009/APX3330 inhibits

Prostate cancer is one of the leading causes of cancer-related death in American men, and challenges remain in targeting key drivers of the aggressive phenotype despite recent advances in prostate cancer treatment. Androgen deprivation therapies and microtubule-targeting agents prolong survival but resistance to these therapeutics is inevitable. It is thought that this resistance is driven in part by aberrant survival signaling and the induction of survival proteins which allows for the cancer to evade cell death [41–42]. Survivin is a bifunctional protein that has been shown to be overexpressed in a number of different cancers including prostate cancer. Survivin has anti-apoptotic and pro-proliferative functions in cancer cells. Inhibition of survivin is a logical therapeutic strategy, however directly targeting survivin has been difficult. In this study, we took a novel approach to survivin targeting; we provide evidence that targeting the redox-signaling regulator APE1/Ref-1 with small molecule inhibitors effectively suppresses survivin protein levels and inhibits cell proliferation (Figure 8).

APE1/Ref-1 is a multifunctional protein that was initially discovered as an enzyme in the base excision repair (BER) pathway, but has also emerged as a redox-signaling regulator of a number of transcription factors known to be involved in cancer, namely NFĸB, AP-1, HIF1a, and STAT3 [43]. These transcription factors have been shown to be important in the initiation and progression of prostate cancer, as well as other cancers. [44–46] In this way, inhibiting the redox activity of APE1/Ref-1 effectively targets multiple different pathways at once and may therefore represent an advantageous therapeutic strategy [47].

The data presented in our studies further support the rationale for APE1/Ref-1 as a viable target in prostate cancer. Our results indicate that APE1/Ref-1 and survivin are expressed in human primary and metastatic tumors as previously reported by Kelley et al. [48]. APE1/Ref-1 and survivin was found to be primarily nuclear localized but cytoplasmic staining was present in the tumors. This pattern was also exhibited in the prostatic cell lines with the cancerous cell lines being positive for APE1/Ref-1 and survivin cytoplasmic localization. Survivin cytoplasmic expression has been associated with poor prognosis in breast, lymphoma, non-small cell lung, live, gastric, ovarian and colorectal cancer [49]. In lung, ovarian, thyroid, and breast cancers, APE1/Ref-1 cytoplasmic distribution has been associated with a higher aggressiveness of the tumor [50].

Similar to other cancer cell lines, we found that APE1/Ref-1 siRNA knockdown decreased cell proliferation and survivin protein levels. Additionally, we demonstrate that inhibition of APE1/Ref-1 redox activity halts prostate cancer cell growth and induces G1 cell arrest in prostate cancer, consistent with recent reports in other cancers. APE1/Ref-1 is crucial in moving cells from G1 to S, and redox inhibition induces key cyclin-dependent kinase inhibitors (CDKi’s) like p21 and p27 [51–52]. This is a translationally relevant finding, as the first-generation APE1/Ref-1 small molecule inhibitor APX3330 used in this study is now approved for phase 1 clinical trials [Investigational New Drug (IND) application number 125360]. APX3330, and the second generation molecule APX2009, is known to bind to APE1/Ref-1 in the redox active region of the protein, cause unfolding of the APE1/Ref-1 protein and block the redox active cysteine 65 from functioning, thus effectively inhibiting its transcriptional regulatory activity of growth signaling pathways [53–56]. APX3330 has been shown to decrease cell proliferation in other cancers including pancreatic and ovarian, and here we show it has similar affects in prostate cancer. Even though cell cycle arrest is what we primarily observed, cell death may occur at later time points and more studies are needed to determine this.

A noteworthy point of these findings is that benign prostate cells also respond to redox-selective APE1/Ref-1 inhibition. This may suggest that benign cells of the prostate could also be affected by Ref-1 inhibition, however even though the E7-transformed cells are considered noncancerous, they still contain a defect in cell cycle regulation due to immortalization via the E7 gene transfection. In other organ systems, normal differentiated cells have not been responsive to Ref-1 inhibition. [57] In fact, APX3330 and APX2009 show a protective effect in neurons, the mechanism of which is still under active investigation [57–59]. While we do not know what effect these inhibitors would have on normal cells in the prostate, *in vivo*, given all the previous preclinical and clinical toxicology studies showing no effects on normal cells, we would not anticipate normal prostate cells to be any different. APX3330 was previously developed for a hepatitis indication and used in over 400 patients with no toxicity. The levels obtained by Eisai in their clinical trials were between 50 and 150 uM in blood such that our dosing is at or below the obtainable level. The proposed APX3330 Phase 1 clinical trial in cancer patients which will be initiating soon will start at a dose that is above levels used in these studies.

Survivin is known to be differentially regulated in various tissues and in response to external stimuli [60]. Survivin is transcriptionally regulated by a number of transcriptional activators including STAT3 and NFĸB. NFĸB-driven survivin protein expression was interrogated here due to our observation that APE1/Ref-1 inhibition is effective in PC-3 cells despite their lacking the gene coding for STAT3. All four cell lines express functional NFĸB signaling [61]. We provide evidence that survivin is at least partially transcriptionally regulated by NFĸB. Upon APE1/Ref-1 redox inhibition, survivin mRNA levels are reduced and IP experiments demonstrate a strong interaction between APE1/Ref-1 and NFĸB subunit p65. Following modulation of APE1/Ref-1 signaling with APX2009 and NFĸB inhibitor PDTC, NFĸB signaling was decreased by 2-fold as assessed by NFĸB-driven luciferase activity. In addition to this decrease in NFkB activity, we also demonstrated that survivin protein levels are reduced by APX2009 (95% reduction) and PDTC (67% reduction) [62]. This reduction in survivin protein levels could occur due to diminished paracrine signaling factors such as, IL-6 or IL-8, which activate NFĸB. It is also possible that APX2009 and PDTC disrupt p65 nuclear trafficking/DNA binding [63]. Further experiments are needed to elucidate this. Nevertheless, our data do not preclude that multiple transcription factors could be contributing to survivin protein levels, and future studies will be directed at carefully assessing the role of each potential transcriptional activator in APE1/Ref-1-mediated prostate cancer cell growth and survivin expression.

In summary, our data indicate that APE1/Ref-1’s redox function plays a role in regulating the proliferative capacity of PCa cells by perturbation of NFĸB transcriptional activity and survivin protein levels in human prostate cancer cell lines and *in vivo* in tumors. Survivin plays an important role in prostate cancer survival, progression and therapeutic resistance. Thus, inhibition of APE1/Ref-1’s redox function in combination with the current therapeutics like docetaxel or cabazataxel may prove to be novel treatment strategy in advanced prostate cancer.

## MATERIALS AND METHODS

### Ethics statement

Investigation has been conducted in accordance with the ethical standards and according to the Declaration of Helsinki and according to national and international guidelines and has been approved by the authors’ institutional review board.

### Cell lines

PC-3, LNCaP and C4-2 prostate cancer cell lines were purchased from and authenticated by the ATCC (Manassas, VA). E7 prostate epithelial cells were generated in the lab from human benign prostate tissue using the method published by Schwarze et al., Dr. David Jarrard, Department of Urology, University of Wisconsin Madison [64]. All cell lines were maintained at 37°C in 5% CO2 and grown in RPMI (Corning: Manassas, VA) with 5% Fetal Bovine Serum (HyClone: Logan, UT).

### Drugs

APX3330, which is also called E3330 [61], was synthesized as previously described [65]. APX2009 was a kind gift from Apexian Pharmaceuticals LLC (Indianapolis, IN). Synthesis and description of APX2009 and RN7-58 has been previously described [65–66]. PDTC (Ammonium pyrrolidinedithiocarbamate) (ab141406) was obtained from Dr. Tao Lu (Indianapolis, IN) who purchased it from Abcam (Cambridge, MA).

### Immunofluorescence

Human prostate specimens or C4-2 xenograft tumors were fixed in 10% formalin, processed routinely, embedded in paraffin, and serially sectioned at 5 µm via microtome. Tissues were subjected to heat-induced antigen retrieval in 10 mM citrate buffer (citrate buffer stock solution of monohydrate-free acid citric acid, sodium citrate dehydrate, pH 6.0) for 10 minutes followed by 10 minute rest. Sections were blocked at room temperature with a bovine serum albumin (BSA)-Donkey serum mixture for 2 hours and incubated with primary antibody overnight at 4°C. Primary antibodies and dilutions included rabbit survivin (1:100, Cell Signaling Technologies), mouse APE1/Ref-1 (1:200, Novus Biologicals), rabbit BrdU (1:200, Cell Signaling Technologies), and mouse PanCK (1:200, Cell Signaling Technologies). Sections were washed with 1X PBS (Phosphate-buffered saline)-Tween and incubated with IgG Alexa 488 and IgG Alexa 594-conjugated secondary antibody against rabbit or mouse for 1 hour at room temperature (1:200, Invitrogen), followed by 10 minutes incubation with Hoechst 33258 nuclear stain (1µg/ml). Tissues were washed with 1× PBS-Tween and water and then covered with an aqueous medium/glass coverslips. The sections were analyzed by immunofluorescence.

### Human specimens

Human prostate specimens (*n* = 12) were obtained with appropriate minimal risk institutional review board approval according to the approval and guidelines at Indiana University School of Medicine. Sections were cut from pre-existing paraffin-embedded human prostate tissues obtained as part of a prostatectomy or from prostate specimens removed collaterally from bladder cancer patients undergoing cystoprostatectomy as control human specimens. These controls were age-matched to the prostate cancer specimens and were verified by record to be naïve for pretreatment with Bacillus Calmette-Guérin (BCG) because these patients had presented first with muscle invasive bladder cancer. The controls were verified by pathology to be void of prostate cancer, BPH, or prostatitis. All human specimens were stained with survivin and APE1/Ref-1 antibodies for immunofluorescence, as described above.

### Immunoblotting

Prostate cells were homogenized in lysis buffer containing protease inhibitor (150 mM NaCl, 10 mM tris, 1 mM EDTA, 1 mM benzenesulfonyl fluoride, and 10 µg/ml each of aprotinin, bestatin, L-luecine, and pepstatin A) and 1% Triton X-100. Total protein concentration was determined by BCA (bicinchoninic acid) assay (Pierce, Rockford, IL). 10 µg/well of Protein were resolved by electrophoresis in 4–15% gradient polyacrylamide gels (Bio-Rad Laboratories). Proteins were transferred to polyvinylidene difluoride (PVDF) membranes, blocked for 24 hours [(10% Dry milk, 5% BSA, .05% NaN_3_) in 1xPBS( 2.7 mM KCl, 1.5 mM KH_2_PO_4_, 136 mM NaCl, 8 mM Na_2_HPO_4_)-Tween 20] and incubated overnight with one of the following primary antibodies: mouse β-actin (1:2500, ThermoFisher Scientific), mouse APE1/Ref-1 (1:1000, Novus Biologicals), rabbit survivin (1:500, Cell Signaling Technologies), rabbit Bcl-2 (1:500, Cell Signaling Technologies), rabbit Mcl-1 (1:500, Cell Signaling Technologies), rabbit Cleaved Caspase 3 (1:250, Cell Signaling Technologies), rabbit Total Caspase 3 (1:1000, Cell Signaling Technologies), rabbit Cyclin B1 (1:500, Cell Signaling Technologies), Cdc2 (1:1000, Cell Signaling Technologies) and rabbit GAPDH (1:1000, Cell Signaling Technologies). After blots were washed 6 times with PBS-Tween, blots were incubated with donkey antibody against rabbit or mouse immunoglobulin G conjugated to horseradish peroxidase for 1 hour (1:10,000 dilution, Pierce) in nonfat dry milk, 1× PBS, and .05% Tween 20. Peroxidase activity was detected via Pico/ West-Femto chemiluminescence reagent (Pierce). Photo images were analyzed by densitometry.

### Methylene blue assay (cell proliferation)

Prostate cells were seeded 1,000-5000 per well (cell line/experiment-dependent) and treated with either APX3330, APX2009 or RN7-58 for 5 days. Media was then removed and cells were fixed with methanol for 10 minutes and stained with 100 µL of 0.05% of methylene blue (LC16920-1 diluted in 1× PBS) for 1 hour. The cells were then washed 3× with water and allowed to air dry overnight. 100 µL’s of 0.5N HCl was added to each well to dissolve the methylene blue stain and absorbance (@630nm) was measured via spectrophotometry. The percent viabilities, normalized to DMSO control, were graphed and IC_50_ concentrations determined. DMSO control was not significantly different from media alone cells.

### Reverse transcription-polymerase chain reaction (RT-PCR)

RNA isolation was performed using RNeasy Mini Kit (Qiagen). 10 nanograms of total RNA was reverse transcribed using Superscript III One-Step RT-PCR System (ThermoFisher Scientific). Real-time PCR was performed using the TaqMan Gene Expression Assay (BIRC5 (Hs04194392_s1) and HPRT1 (Hs02800695_m1), ThermoFisher Scientific) and Applied Biosystems 7500 Fast Real-Time PCR System.

### Co-immunoprecipitation

Samples were co-immunoprecipitated using the Pierce Co-IP kit (Thermo Scientific). Additionally, the cells were washed twice with 1× PBS and the proteins were cross-linked using DTBP (Thermo Scientific, 5 mm, for 30 min on ice). DTBP was quenched by washing with cold inactivation buffer (100 mm Tris-HCl, pH 8, 150 mm NaCl) and 1XPBS. Cells were then lysed and the lysates added to columns and after extensive washing, the bound proteins were eluted and prepared for immunoblot analysis.

### Luciferase assay

C4-2 cells were co-transfected with constructs containing luciferase driven by NFκB (pLuc-MCS with NFκB responsive promoter; P0athDetect cis-Reporting Systems, Stratagene, La Jolla, Ca) and a Renilla luciferase control reporter vector pRL-TK (Promega Corp., Madison, WI) at a 20:1 ratio by using Effectene Transfection Reagent (Qiagen; Valencia, CA). After 16 hours, cells were treated with increasing concentrations of APX2009 or PDTC in serum free media for 24 hours. Firefly and Renilla luciferase activities were assessed by using the Dual Luciferase Reporter Assay System (Promega Corp.). Renilla luciferase activity was used for normalization and all transfection experiments were performed in triplicate and repeated 3 times in independent experiments.

### Propidium iodide (PI) cell cycle analysis

PC-3 and C4-2 cells were treated with APX2009 (9 and 14 µM, respectively) for 48 hours. 500,000 cells were then aliquoted for cell cycle analysis and 0.1 mg/ml Propidium Iodide and 0.6% NP-40 PBS stain wash was added to the tubes. The cells were then centrifuged at 1900 rpms for 10 minutes with the brake on low and then decanted and blotted. RNAase and stain wash were added and cells incubated on ice for 30 minutes. Propidium Iodide intensity was measured via flow cytometry.

### *In vivo* subcutaneous tumor

2 × 10^6^ C4-2 cells were subcutaneously implanted in the hind flank of male athymic nude mice using a 100μl volume of 50:50 solution of Matrigel: RPMI medium. When tumor volumes reached 150 –200 mm^3^, the animals were treated with 25 mg/kg IP APX2009 or vehicle (Propylene Glycol Kolliphor HS15 Tween 80 (PKT)) every 12 hours for 5 days. BrdU was injected into the animals 2 hours prior to sacrifice and tumor tissues were analyzed for survivin levels (immunofluorescence and immunoblotting) and BrdU incorporation (immunofluorescence).

### siRNA transfection

All siRNA transfections were performed using the HiPerfect Transfection Reagent (Qiagen) protocol. Post-transfection C4-2 cells (1,000 per well) and PC-3 cells (1,500 per well) were replated in a 96 well plate and fixed daily up to 6 days and methylene blue assay was performed. Samples for immunoblotting were collected 72 hours post transfection of cancer cells with APE1/Ref-1 siRNA and scrambled siRNA control. Prevalidated APE1/Ref-1 siRNA (siAPE1 #2) was purchased from LifeTech (#s1446).

### Statistics

Summary statistics are presented using the mean, median, and SD. Either a Student’s *t*-test or ANOVA test was performed to compare the groups as appropriate. Statistical significance was assessed at the *p* < 0.05.

## SUPPLEMENTARY MATERIALS FIGURES


