# Supplementary material for: APE1/Ref-1 redox-specific inhibition decreases survivin protein levels and induces cell cycle arrest in prostate cancer cells
Source: Oncotarget. 2017 Dec 13;9(13):10962–77. doi: 10.18632/oncotarget.23493 (PMC5834255; doi:10.18632/oncotarget.23493)
Supplement: Supplementary file 1 [file oncotarget-09-10962-s001.pdf]

APE1/Ref-1 redox-specific inhibition decreases survivin protein levels and induces cell cycle arrest in prostate cancer cells

SUPPLEMENTARY MATERIALS

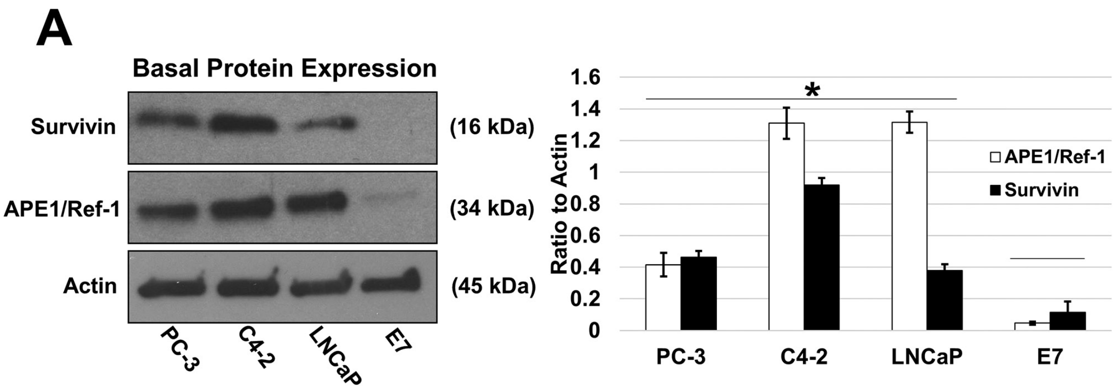

Supplementary Figure 1: APE1/Ref-1 and survivin are overexpressed in prostate cancer cells. Immunoblot example of basal survivin and APE1/Ref-1 protein levels between the prostatic cell lines. Representative of three determinations with densitometry quantification. $N = 3$, *-denoting $p < 0.05$ (PC-3, C4-2 and LNCaP vs. E7) as assessed by ANOVA.

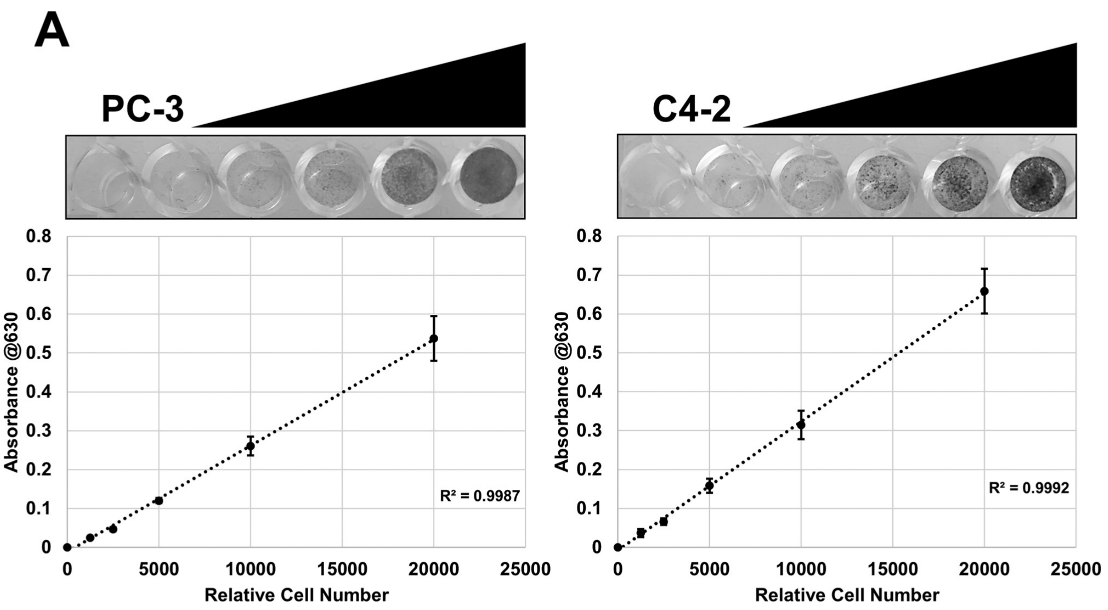

Supplementary Figure 2: Methylene blue assay. PC-3 and C4-2 cells were seeded 1,000–20,000 per well. Media was then removed and cells were fixed with methanol for 10 minutes and stained with 100 µL of 0.05% of methylene blue (LC16920-1 diluted in 1× PBS) for 1 hour. The cells were then washed 3× with water and allowed to air dry overnight. Representative pictures were taken. 100 µL's of 0.5N HCl was added to each well to dissolve the methylene blue stain and absorbance (@630 nm) was measured via spectrophotometry (Figure 10). Equations were derived from these trend lines and used to calculate relative cell number in subsequent experiments.

A

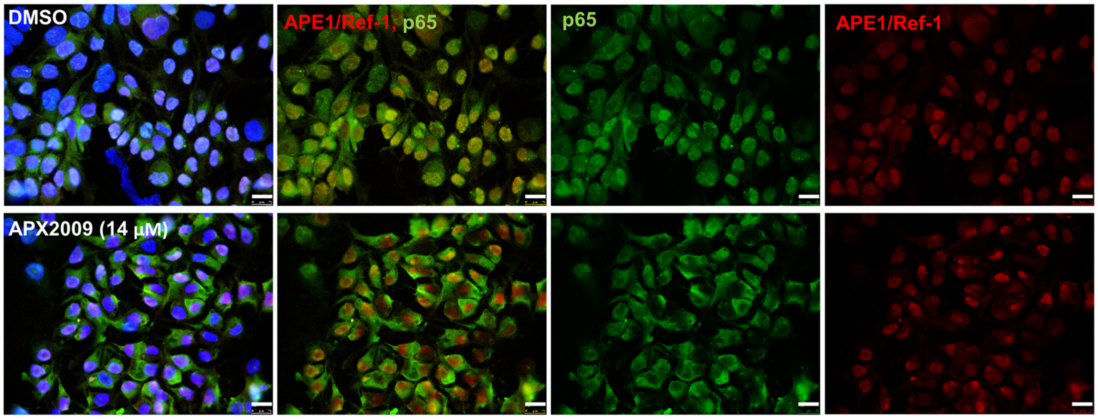

Supplementary Figure 3: Cellular localization of NFκB is altered upon APE1/Ref-1 redox inhibition. C4-2 cells were treated with either DMSO or 14 μM APX2009 for 48 hours and then fixed for immunofluorescence (p65 = Green and APE1/Ref-1 = Red). p65 and APE1/Ref-1 were found to be co-localized in the nucleus. However upon treatment with APX2009, p65 nuclear localization was diminished.
